# Supplementary material for: Virtual Screening and Quantum Chemistry Analysis for SARS-CoV-2 RNA-Dependent RNA Polymerase Using the ChEMBL Database: Reproduction of the Remdesivir-RTP and Favipiravir-RTP Binding Modes Obtained from Cryo-EM Experiments with High Binding Affinity
Source: Int J Mol Sci. 2022 Sep 20;23(19):11009. doi: 10.3390/ijms231911009 (PMC9570209; doi:10.3390/ijms231911009)
Supplement: Supplementary file 1 [file ijms-23-11009-s001.zip › MTsuji-TablesS1-S2.pdf]

**Supporting Information**

# Virtual screening and quantum chemistry analysis for SARS-CoV-2 RNA-dependent RNA polymerase using the ChEMBL database: Reproduction of the remdesivir-RTP and favipiravir-RTP binding modes obtained from cryo-EM experiments with high binding affinity

Motonori Tsuji [1,*]

[1] Institute of Molecular Function, 2-105-14 Takasu, Misato-shi, Saitama 341-0037, Japan

motonori@molfunction.com

**Contents**

**Table S1.** Potential anti-SARS-CoV-2 drugs obtained from rDock virtual screening of the ChEMBL database.
**S2-S11**

**Table S2.** Hit compounds obtained by combining the AutoDock Vina and rDock virtual screening results using the ChEMBL database.
**S11-S17**

**Table S1.** Potential anti-SARS-CoV-2 drugs obtained from rDock virtual screening of the ChEMBL database.

| ChEMBL ID | Synonym | Status | Target | Action | rDock Score (kcal/mol) | AutoDock Vina Score (kcal/mol) |
|---|---|---|---|---|---|---|
| CHEMBL2106851 | NICOMORPHINE | | Opioid receptor mu 1 | Analgesic | −61.521 | −10.2 |
| CHEMBL1371412 | TIOXIDAZOLE | | Prelamin-A/C | Anthelmintic | −63.708 | −6.7 |
| CHEMBL547 | ISOTRETINOIN | Approved | Retinoic acid receptor (RAR) agonist | Anti-acne | −60.736 | −7.3 |
| CHEMBL23771 | TIXANOX | | Aldose reductase | Anti-allergic | −60.962 | −8.1 |
| CHEMBL296419 | ASTEMIZOLE | Approved | H1 receptor antagonist | Anti-allergic | −65.952 | −9.2 |
| CHEMBL61301 | TECASTEMIZOLE | | Histamine receptor H1 antagonist | Anti-allergic | −85.018 | −7.7 |
| CHEMBL2104845 | ONTAZOLAST | | Eukotriene receptor antagonist | Antiasthmatic | −63.044 | −8.2 |
| CHEMBL501122 | CEFTAROLINE FOSAMIL | Approved | Penicillin binding protein | Antibacterial | −85.659 | −10.3 |
| CHEMBL161 | CEFTRIAXONE | Approved | Penicillin binding protein | Antibacterial | −67.839 | −10.0 |
| CHEMBL2110979 | PIRBENICILLIN | | Penicillin binding protein | Antibacterial | −64.577 | −9.7 |
| CHEMBL2110884 | CETOCYCLINE | | | Antibacterial | −66.912 | −9.6 |
| CHEMBL44354 | CEFTAZIDIME | Approved | Penicillin binding protein | Antibacterial | −60.388 | −9.0 |
| CHEMBL1237124 | MECLOCYCLINE | | 30S ribosomal subunit | Antibacterial | −62.029 | −9.0 |
| CHEMBL1697786 | CEFUROXIME PIVOXETIL | | Penicillin binding protein | Antibacterial | −66.707 | −9.0 |
| CHEMBL1433 | DOXYCYCLINE | Approved | 30S ribosomal subunit | Antibacterial | −63.212 | −8.9 |
| CHEMBL1583 | BACAMPICILLIN | Approved | Monoamine oxidase A | Antibacterial | −67.654 | −8.7 |
| CHEMBL1524273 | PHTHALYLSULFATHIAZOLE [1] | Approved | Dihydropteroate synthase | Antibacterial | −96.910 | −8.6 |
| CHEMBL1109 | SULFAPHENAZOLE | Approved | Dihydropteroate synthase | Antibacterial | −89.002 | −8.2 |
| CHEMBL2104908 | SULFAZAMET | | | Antibacterial | −94.032 | −8.2 |
| CHEMBL1484857 | SUCCINYLSULFATHIAZOLE | | Dihydropteroate synthase | Antibacterial | −92.546 | −7.9 |
| CHEMBL1167 | SPECTINOMYCIN | Approved | 30S ribosomal subunit | Antibacterial | −60.601 | −7.7 |
| CHEMBL1355299 | SULFAETHIDOLE [1] | | Putative fructose-1,6-bisphosphate aldolase | Antibacterial | −77.373 | −7.6 |
| CHEMBL1191 | SULFAMETHIZOLE [1] | Approved | Dihydropteroate synthase | Antibacterial | −85.152 | −7.5 |

| | | | | | | |
|---|---|---|---|---|---|---|
| CHEMBL453 | SULFISOXAZOLE [1] | Approved | Dihydropteroate synthase | Antibacterial | −93.518 | −7.4 |
| CHEMBL2105399 | SULFAMOXOLE [1] | | Dihydropteroate synthase | Antibacterial | −75.727 | −7.4 |
| CHEMBL2107088 | SULFASOMIZOLE | | | Antibacterial | −85.069 | −7.3 |
| CHEMBL437 | SULFATHIAZOLE | Approved | Dihydropteroate synthase | Antibacterial | −95.889 | −7.2 |
| CHEMBL4594290 | TANIBORBACTAM | Phase1 | beta-Lactamase inhibitor | Antibacterial | −60.599 | nd [4] |
| CHEMBL3544919 | CIRAPARANTAG | Phase2 | | Anticoagulant | −64.728 | −8.7 |
| CHEMBL549671 | SABELUZOLE | | | Anticonvulsant | −79.056 | −9.0 |
| CHEMBL310160 | BIPENAMOL | | Trypanothione reductase | Antidepressant | −69.331 | −6.5 |
| CHEMBL1770248 | ERTUGLIFLOZIN | Approved | SGLT-2 inhibitor | Anti-diabetic | −68.404 | −9.1 |
| CHEMBL450117 | GLYPROTHIAZOL | | Monoamine oxidase A | Anti-diabetic | −78.899 | −7.6 |
| CHEMBL2107408 | GLYBUZOLE [1] | | | Anti-diabetic | −89.891 | −7.3 |
| CHEMBL94087 | GLYBUTHIAZOL [1] | | | Anti-diabetic | −78.713 | −7.3 |
| CHEMBL550348 | DEFERASIROX | Approved | | Antidote | −68.372 | −8.9 |
| CHEMBL1950289 | TANZISERTIB [1] | Phase2 | c-Jun N-terminal kinase inhibitor | Antifibrotic | −86.574 | −9.5 |
| CHEMBL4297591 | FOSMANOGEPIX | Phase2 | Glucosaminylphosphatid ylinositol acyltransferase | Antifungal | −65.501 | −9.3 |
| CHEMBL333325 | FOSRAVUCONAZOLE | Phase2 | Sterol 14alpha-demethylase | Antifungal | −63.940 | −8.9 |
| CHEMBL2105391 | PROTIOFATE | | | Antifungal | −60.916 | −6.2 |
| CHEMBL2105913 | BARMASTINE | | Histamine receptor H1 | Antihistaminic | −93.233 | −9.2 |
| CHEMBL539423 | SPARSENTAN | Phase3 | Angiotensin II receptor type 1 | Anti-hypertensive | −84.211 | −9.3 |
| CHEMBL2111148 | TIPENTOSIN | | | Anti-hypertensive | −63.007 | −8.1 |
| CHEMBL295409 | TIAMENIDINE | | | Anti-hypertensive | −90.127 | −5.6 |
| CHEMBL114586 | SEPIMOSTAT [1] | | Serine protease inhibitor | Anti-inflammatory | −85.231 | −9.7 |
| CHEMBL4297477 | BREPOCITINIB | Phase2 | Janus kinase 1 | Anti-inflammatory | −61.358 | −9.4 |
| CHEMBL1200342 | PARAMETHASONE ACETATE | Approved | Glucocorticoid receptor agonist | Anti-inflammatory | −73.043 | −9.3 |
| CHEMBL2105842 | CORTIVAZOL | Approved | Glucocorticoid receptor agonist | Anti-inflammatory | −74.903 | −9.3 |
| CHEMBL2106520 | HALOPREDONE ACETATE | | Glucocorticoid receptor partial agonist | Anti-inflammatory | −60.970 | −9.2 |
| CHEMBL216981 | NAVARIXIN | Phase2 | Interleukin-8 receptor B | Anti-inflammatory | −120.782 | −8.8 |
| CHEMBL1385514 | DICHLORISONE ACETATE | | Glucocorticoid receptor agonist | Anti-inflammatory | −72.408 | −8.1 |

| | | | | | | |
|---|---|---|---|---|---|---|
| CHEMBL2105059 | MORNIFLUMATE | | | Anti-inflammatory | −62.266 | −8.1 |
| CHEMBL1413176 | NIFENAZONE | | Menin/Histone-lysine N-methyltransferase MLL | Anti-inflammatory | −61.214 | −7.6 |
| CHEMBL2104561 | ELTENAC [1] | | | Anti-inflammatory | −71.821 | −6.7 |
| CHEMBL3809489 | BEMCENTINIB | Phase2 | Receptor tyrosine kinase inhibitor | Anti-neoplastic | −97.163 | −11.2 |
| CHEMBL3989868 | TUCATINIB | Approved | erb-B2 receptor tyrosine kinase 2 | Anti-neoplastic | −78.569 | −11.0 |
| CHEMBL3904602 | LEROCICLIB | Phase1 | Cyclin-dependent kinase (CDK) inhibitor | Anti-neoplastic | −64.184 | −10.7 |
| CHEMBL4582651 | PRALSETINIB | Phase3 | Replicase polyprotein 1ab | Anti-neoplastic | −90.662 | −10.6 |
| CHEMBL4130229 | VODOBATINIB | Phase2 | BCR-ABL | Anti-neoplastic | −65.358 | −10.3 |
| CHEMBL3889654 | LAROTRECTINIB | Approved | Tropomyosin receptor kinase inhibitor | Anti-neoplastic | −68.149 | −10.1 |
| CHEMBL3218576 | COPANLISIB | Approved | Phosphatidylinositol 3-kinase inhibitor | Anti-neoplastic | −71.829 | −10.0 |
| CHEMBL3301622 | GILTERITINIB | Approved | Tyrosine-protein kinase receptor UFO | Anti-neoplastic | −64.290 | −10.0 |
| CHEMBL1908391 | MASITINIB | Phase3 | Dual specificty protein kinase CLK1 | Anti-neoplastic | −90.568 | −10.0 |
| CHEMBL522892 | DOVITINIB | Phase3 | Vascular endothelial growth factor receptor 2 | Anti-neoplastic | −60.745 | −10.0 |
| CHEMBL2107823 | GANDOTINIB | Phase2 | Janus kinase (JAK) inhibitor | Anti-neoplastic | −96.129 | −10.0 |
| CHEMBL1421 | DASATINIB | Approved | Tyrosine kinase inhibitor | Anti-neoplastic | −99.368 | −9.9 |
| CHEMBL3265032 | ENTOSPLETINIB | Phase2 | Tyrosine-protein kinase SYK | Anti-neoplastic | −64.693 | −9.9 |
| CHEMBL1091644 | LINSITINIB | Phase3 | Insulin-like growth factor 1 receptor antagonist | Anti-neoplastic | −62.711 | −9.8 |
| CHEMBL4297188 | MIRANSERTIB [2] | Phase2 | AKT serine/threonine kinase inhibitor | Anti-neoplastic | −67.950 | −9.6 |
| CHEMBL4594275 | GUSACITINIB | Phase2 | Janus kinase 1 | Anti-neoplastic | −63.792 | −9.5 |
| CHEMBL31965 | CANERTINIB | Phase3 | Epidermal growth factor receptor erbB1 | Anti-neoplastic | −61.274 | −9.4 |
| CHEMBL3545369 | EPACADOSTAT | Phase3 | Indoleamine 2,3-dioxygenase inhibitor | Anti-neoplastic | −93.000 | −9.4 |
| CHEMBL1287853 | FEDRATINIB | Approved | Janus kinase (JAK) inhibitor | Anti-neoplastic | −65.415 | −9.3 |
| CHEMBL4297190 | SURUFATINIB | Phase3 | Tyrosine kinase inhibitor | Anti-neoplastic | −61.578 | −9.3 |
| CHEMBL2103830 | FOSTAMATINIB | Approved | Tyrosin kinase inhibitor | Anti-neoplastic | −61.766 | −9.2 |
| CHEMBL252164 | LUMINESPIB | Phase2 | Hsp 90 inhibitor | Anti-neoplastic | −60.834 | −9.2 |

| | | | | | | |
|---|---|---|---|---|---|---|
| CHEMBL4297595 | LISAVANBULIN | Phase1 | Tubulin polymerization inhibitor | Anti-neoplastic | −61.208 | −9.2 |
| CHEMBL25336 | BISANTRENE [1] | Phase3 | RPMI-8226 | Anti-neoplastic | −101.755 | −9.1 |
| CHEMBL2103842 | VARLITINIB [1] | Phase2 | Receptor protein-tyrosine kinase erbB-2 | Anti-neoplastic | −72.031 | −9.1 |
| CHEMBL3989970 | MAVELERTINIB | Phase2 | Epidermal growth factor receptor erbB1 | Anti-neoplastic | −76.890 | −9.1 |
| CHEMBL2110669 | OMTRIPTOLIDE | | DNA | Anti-neoplastic | −62.258 | −8.9 |
| CHEMBL1094636 | NIRAPARIB | Approved | PARP inhibitor | Anti-neoplastic | −60.508 | −8.8 |
| CHEMBL3353410 | OSIMERTINIB | Approved | Epidermal growth factor receptor erbB1 | Anti-neoplastic | −62.621 | −8.8 |
| CHEMBL428690 | ALVOCIDIB | Phase3 | Cyclin-dependent kinase (CDK) inhibitor | Anti-neoplastic | −75.444 | −8.8 |
| CHEMBL4558324 | LAZERTINIB | Phase1 | Epidermal growth factor receptor | Anti-neoplastic | −61.508 | −8.7 |
| CHEMBL3422109 | AVANBULIN | | Tubulin polymerization inhibitor | Anti-neoplastic | −84.936 | −8.6 |
| CHEMBL435191 | EDOTECARIN | Phase3 | Topoisomerase I inhibitor | Anti-neoplastic | −63.426 | −8.5 |
| CHEMBL271068 | ROSABULIN | Phase1 | | Anti-neoplastic | −60.832 | −8.5 |
| CHEMBL2105946 | AMBOMYCIN | | | Anti-neoplastic | −63.695 | −8.0 |
| CHEMBL3544911 | PREXASERTIB [2] | Phase2 | Checkpoint kinase inhibitor | Anti-neoplastic | −91.788 | −7.9 |
| CHEMBL4303782 | ZOTATIFIN [2] | Phase1 | RNA helicase inhibitor | Anti-neoplastic | −61.715 | −7.2 |
| CHEMBL476 | DACARBAZINE | Approved | DNA, RNA | Anti-neoplastic | −63.025 | −5.6 |
| CHEMBL459505 | TALAROZOLE | Phase2 | Cytochrome P450 family 26 subfamily A member 1 | Antipsoriatic | −93.312 | −7.9 |
| CHEMBL53950 | INCADRONIC ACID | | Farnesyl diphosphate synthase | Antiresorptive | −63.453 | −6.6 |
| CHEMBL1350 | TILUDRONIC ACID | | | Antiresorptive | −60.652 | −6.5 |
| CHEMBL14249 | ADENOSINE TRIPHOSPHATE [3] | Phase2 | P2Y purinoceptor 1 | Antivertigo | −62.435 | −8.8 |
| CHEMBL473882 | ENTECAVIR TRIPHOSPHATE [3] | | HBV reverse transcriptase | Antiviral | −67.745 | −9.7 |
| CHEMBL3818050 | FAVIPIRAVIR-RTP [2,3] | | Influenza A virus RNA-directed RNA polymerase | Antiviral | −67.292 | −9.1 |
| CHEMBL2016761 | RDV-TP [3] | | Hepatitis C virus | Antiviral | −62.583 | −8.9 |
| CHEMBL4594375 | PENCICLOVIR TRIPHOSPHATE [3] | | Herpesvirus DNA polymerase inhibitor | Antiviral | −66.854 | −8.7 |
| CHEMBL290507 | ENVIRADENE | | | Antiviral | −60.236 | −8.4 |
| CHEMBL1230 | LAMIVUDINE | | HIV reverse | Antiviral | −63.543 | −8.2 |

| | | | | | | |
|---|---|---|---|---|---|---|
| | TRIPHOSPHATE [3] | | transcriptase inhibitor | | | |
| CHEMBL3039503 | LOMIBUVIR | Phase2 | HCV NS5B polymerase | Antiviral | −70.808 | −8.1 |
| CHEMBL515408 | MARIBAVIR | Phase3 | HCMV protein kinase | Antiviral | −78.670 | −8.0 |
| CHEMBL1162433 | ADEFOVIR DIPHOSPHATE | | Reverse transcriptase inhibitor | Antiviral | −61.475 | −7.9 |
| CHEMBL1214598 | UMIFENOVIR | Phase3 | | Antiviral | −61.709 | −7.6 |
| CHEMBL1538 | TENOFOVIR DISOPROXIL | Approved | HBV reverse transcriptase | Antiviral | −61.255 | −7.5 |
| CHEMBL2111023 | NISBUTEROL | | | Bronchodilator | −63.683 | −7.6 |
| CHEMBL436559 | CARIPORIDE | | Na+/H+ antiport inhibitor | Cardioprotectant | −66.526 | −7.3 |
| CHEMBL1014 | CANDESARTAN CILEXETIL | Approved | Angiotensin II receptor antagonist | Cardiovascular | −65.673 | −10.3 |
| CHEMBL539697 | DABIGATRAN ETEXILATE | Approved | Thrombin inhibitor | Cardiovascular | −71.432 | −9.2 |
| CHEMBL1697762 | NICERITROL | | | Cardiovascular | −64.854 | −8.9 |
| CHEMBL282724 | SITAXENTAN | Approved | Endothelin receptor type A antagonist | Cardiovascular | −84.495 | −8.6 |
| CHEMBL2107326 | DASANTAFIL | | Phosphodiesterase V inhibitor | Cardiovascular | −78.105 | −8.3 |
| CHEMBL2105424 | ROMIFIDINE | | Adrenergic receptor agonist | Cardiovascular | −68.171 | −7.0 |
| CHEMBL35505 | DIHYDRALAZINE | | | Cardiovascular | −61.789 | −6.9 |
| CHEMBL1079 | TIZANIDINE [1] | | alpha-Adrenergic receptor agonist | Cardiovascular | −90.626 | −6.6 |
| CHEMBL19236 | MOXONIDINE | Approved | Adrenergic receptor alpha-1 | Cardiovascular | −78.408 | −6.3 |
| CHEMBL1197578 | CYPROQUINATE | | | Coccidiostat | −62.345 | −7.5 |
| CHEMBL2368925 | DOLASETRON | Approved | Serotonin 5-HT3 receptor antagonist | Gastrointestinal | −61.293 | −8.5 |
| CHEMBL2105496 | SUFOTIDINE | | Histamine receptor H2 antagonist | Gastrointestinal | −81.234 | −8.3 |
| CHEMBL942 | BISACODYL | Approved | Prelamin-A/C | Gastrointestinal | −60.770 | −7.5 |
| CHEMBL1993793 | FANETIZOLE | | Luciferin 4-monooxygenase | Immunomodulator | −64.926 | −7.3 |
| CHEMBL2105568 | LINAROTENE | | | Keratolytic | −60.847 | −9.0 |
| CHEMBL1108 | DROPERIDOL [1] | Approved | Dopamine D2-receptor antagonist | Neuropsychiatric | −62.397 | −9.2 |
| CHEMBL2105747 | TOZADENANT | Phase3 | Adenosine A2A receptor antagonist | Neuropsychiatric | −67.688 | −8.7 |
| CHEMBL398615 | METOPIMAZINE | | Dopamine D2 receptor antagonist | Neuropsychiatric | −61.668 | −8.6 |

| | | | | | | |
|---|---|---|---|---|---|---|
| CHEMBL2107723 | ACOTIAMIDE | Phase3 | Acetylcholinesterase inhibitor | Neuropsychiatric | −61.609 | −8.5 |
| CHEMBL2104165 | CYPRAZEPAM | | | Neuropsychiatric | −64.311 | −7.8 |
| CHEMBL1118 | DESVENLAFAXINE | Approved | alpha-1a Adrenergic receptor | Neuropsychiatric | −62.187 | −6.5 |
| CHEMBL81 | RALOXIFENE | Approved | Selective estrogen receptor modulator | Osteoporosis | −60.788 | −9.2 |
| CHEMBL122079 | LIFARIZINE | | Sodium channel alpha subunits; brain (Types I, II, III) | Platelet aggregation inhibitor | −61.327 | −9.1 |
| CHEMBL153427 | OXAGRELATE | | Phosphodiesterase 1 | Platelet aggregation inhibitor | −71.034 | −7.5 |
| CHEMBL9506 | DEVAZEPIDE | | Cholecystokinin receptor antagonist | Prokinetic | −60.752 | −9.7 |
| CHEMBL2105584 | ULDAZEPAM | | | Sedative-hypnotic | −75.518 | −7.5 |
| CHEMBL2364615 | LUBABEGRON | | | Veterinary | −60.795 | −8.3 |
| CHEMBL3545378 | MK-6592 | Phase1 | Serine/threonine-protein kinase Aurora-B | | −83.808 | −11.0 |
| CHEMBL558752 | RAF-265 | Phase2 | Tyrosine-protein kinase receptor FLT3 | | −67.400 | −10.8 |
| CHEMBL2010872 | CEP-11981 | Phase2 | | | −60.472 | −10.5 |
| CHEMBL575448 | BMS-754807 | Phase2 | Insulin-like growth factor I receptor | | −74.091 | −10.5 |
| CHEMBL4297501 | AFABICIN | Phase2 | | | −67.066 | −10.4 |
| CHEMBL1742417 | POMISARTAN | | | | −60.035 | −10.3 |
| CHEMBL3984425 | IPI-549 | Phase2 | | | −64.454 | −10.2 |
| CHEMBL2346976 | AZD3514 | Phase1 | | | −68.808 | −10.1 |
| CHEMBL3128043 | PF-03758309 | | | | −88.963 | −10.1 |
| CHEMBL4297363 | UCB-1184197 | Phase2 | | | −97.550 | −10.0 |
| CHEMBL1967878 | CENISERTIB | Phase1 | Inhibitor of nuclear factor kappa B kinase epsilon subunit | | −60.855 | −10.0 |
| CHEMBL2359248 | ZEAXANTHIN | Phase3 | Tyrosyl-DNA phosphodiesterase 1 | | −60.523 | −9.9 |
| CHEMBL1908394 | GSK-461364 | Phase1 | Dual specificty protein kinase CLK1 | | −81.373 | −9.9 |
| CHEMBL4521078 | PLX-51107 | Phase1 | Bromodomain-containing protein 4 | | −63.701 | −9.9 |
| CHEMBL2105519 | ACROCINONIDE | | | | −165.537 | −9.9 |
| CHEMBL131854 | POSIZOLID | | | | −67.303 | −9.8 |
| CHEMBL2338675 | FOSDENOPTERIN | | MOCS1 | | −60.184 | −9.8 |

| | | | | | |
|---|---|---|---|---|---|
| CHEMBL415049 | BARASERTIB | Phase3 | | −69.006 | −9.7 |
| CHEMBL1408927 | AMARANTH | | Thyroid hormone receptor beta-1 | −73.728 | −9.7 |
| CHEMBL4594255 | NICEVERINE | | | −62.288 | −9.7 |
| CHEMBL2107729 | ATACIGUAT | Phase2 | | −63.668 | −9.6 |
| CHEMBL482968 | ENMD-2076 | Phase2 | Serine/threonine-protein kinase Aurora-A | −65.872 | −9.6 |
| CHEMBL482767 | SNS-314 | Phase 1 | Inhibitor of nuclear factor kappa B kinase epsilon subunit | −71.806 | −9.6 |
| CHEMBL1320139 | HELIOMYCIN | | Menin/Histone-lysine N-methyltransferase MLL | −66.316 | −9.6 |
| CHEMBL1614646 | SUSALIMOD | | | −61.119 | −9.6 |
| CHEMBL2106914 | PHTHALYLSULFAMETHIZOLE [1] | | | −82.707 | −9.6 |
| CHEMBL587723 | AEE-788 | Phase2 | | −60.105 | −9.5 |
| CHEMBL3989439 | NICOFURATE | | | −60.749 | −9.5 |
| CHEMBL4297397 | QUERCETIN-3'-O-PHOSPHATE | Phase2 | | −62.954 | −9.4 |
| CHEMBL3182444 | MK-5108 [1] | Phase1 | | −79.062 | −9.4 |
| CHEMBL4297470 | MAX-40279 | Phase1 | | −94.922 | −9.4 |
| CHEMBL457077 | SALVIANOLIC ACID A | Phase 1 | | −67.669 | −9.4 |
| CHEMBL1270562 | SALAZOSULFATHIAZOLE | | PH domain leucine-rich repeat-containing protein phosphatase 2 | −88.917 | −9.4 |
| CHEMBL3545215 | BMS-911543 | Phase2 | Phosphodiesterase 4 | −90.978 | −9.3 |
| CHEMBL1939499 | MK-6186 | Phase1 | | −62.056 | −9.3 |
| CHEMBL2178575 | GSK-2239633 | Phase1 | | −94.791 | −9.3 |
| CHEMBL1201369 | GTP [3] | | | −68.191 | −9.3 |
| CHEMBL292187 | ELOPIPRAZOLE | | Dopamine D2 receptor | −62.701 | −9.3 |
| CHEMBL486231 | GS-461203 [3] | | Hepatitis C virus | −64.418 | −9.3 |
| CHEMBL3545241 | AC-430 | Phase1 | Tyrosine-protein kinase JAK1 | −104.425 | −9.2 |
| CHEMBL4297451 | XL-888 | Phase 1 | | −61.289 | −9.2 |
| CHEMBL4297641 | SECLIDEMSTAT | Phase 1 | | −63.420 | −9.2 |
| CHEMBL4594423 | TP-1287 | Phase 1 | | −64.654 | −9.2 |
| CHEMBL4297421 | COH-29 | Phase1 | | −69.684 | −9.1 |
| CHEMBL2057236 | TP-271 | Phase 1 | | −61.562 | −9.1 |
| CHEMBL48880 | ASULACRINE | | | −63.785 | −9.1 |

| | | | | | |
|---|---|---|---|---|---|
| CHEMBL1213492 | GIVINOSTAT | Phase3 | Histone deacetylase 1 | −65.354 | −9.0 |
| CHEMBL318191 | TONABERSAT | Phase2 | | −60.055 | −9.0 |
| CHEMBL495727 | AT-9283 | Phase2 | | −62.873 | −9.0 |
| CHEMBL4297473 | NP-G2-044 | Phase1 | | −61.646 | −9.0 |
| CHEMBL494089 | GSK-690693 | Phase1 | Serine/threonine-protein kinase AKT | −61.825 | −9.0 |
| CHEMBL1090173 | SD-0006 | | MAP kinase p38 alpha | −61.633 | −9.0 |
| CHEMBL2107195 | ISOPREDNIDENE | | | −66.317 | −9.0 |
| CHEMBL142652 | PICEID | Phase2 | | −64.566 | −8.9 |
| CHEMBL253969 | OSI-632 | Phase2 | Tyrosine-protein kinase TIE-2 | −71.745 | −8.9 |
| CHEMBL3545291 | JNJ-39393406 | Phase2 | | −89.518 | −8.9 |
| CHEMBL3786098 | PF-06459988 | Phase2 | Epidermal growth factor receptor erbB1 | −92.876 | −8.9 |
| CHEMBL483321 | CP-724714 | Phase2 | Receptor protein-tyrosine kinase erbB-2 | −63.732 | −8.9 |
| CHEMBL2105118 | LIREQUINIL [1] | | | −61.192 | −8.8 |
| CHEMBL276368 | TIMEGADINE | | | −79.662 | −8.8 |
| CHEMBL4594368 | 2-CHLORODEOXYADENOSINE TRIPHOSPHATE [3] | | | −64.169 | −8.8 |
| CHEMBL4297504 | BELORANIB | Phase3 | | −62.185 | −8.7 |
| CHEMBL4297399 | LY-2608204 [2] | Phase2 | | −67.265 | −8.7 |
| CHEMBL1230607 | PHA-793887 | Phase1 | | −69.733 | −8.7 |
| CHEMBL3544966 | GSK-1059615 | Phase1 | Adaptor-associated kinase | −63.344 | −8.7 |
| CHEMBL4594428 | ASP-5878 | Phase1 | | −88.275 | −8.6 |
| CHEMBL2106007 | COUMETAROL | | | −63.248 | −8.6 |
| CHEMBL2107223 | SULFAGUANOLE | | | −63.498 | −8.6 |
| CHEMBL2058833 | GANAPLACIDE [1] | Phase2 | | −94.555 | −8.5 |
| CHEMBL4594432 | LY-3200882 | Phase1 | | −61.955 | −8.5 |
| CHEMBL1201384 | DDCTP | | Reverse transcriptase | −66.921 | −8.5 |
| CHEMBL1205402 | OXI-4503 | Phase1 | | −60.250 | −8.4 |
| CHEMBL64360 | ENIPORIDE | | Sodium/hydrogen exchanger 1 | −69.610 | −8.4 |
| CHEMBL51483 | GOSSYPOL | Phase3 | | −61.311 | −8.3 |
| CHEMBL1614710 | OSI-930 | Phase1 | Stem cell growth factor receptor | −93.645 | −8.3 |

| | | | | | |
|---|---|---|---|---|---|
| CHEMBL2105528 | BISFENAZONE [1] | | Carboxylesterase | −85.310 | −8.3 |
| CHEMBL2106538 | DOMOPREDNATE | | | −60.337 | −8.3 |
| CHEMBL2106670 | ALESTRAMUSTINE | | | −62.748 | −8.3 |
| CHEMBL2111115 | GLUCOSULFAMIDE | | | −60.309 | −8.3 |
| CHEMBL1742458 | TUVATIDINE | | | −61.010 | −8.1 |
| CHEMBL2106016 | ATRINOSITOL | | | −63.790 | −8.1 |
| CHEMBL338667 | DIPERODON | | Sodium channel alpha subunits; brain (Types I, II, III) | −64.911 | −8.1 |
| CHEMBL3763743 | TTC-352 | | Estrogen receptor alpha | −60.023 | −8.1 |
| CHEMBL1416586 | BENZPIPERYLON | | Inositol monophosphatase 1 | −60.255 | −8.0 |
| CHEMBL2106807 | MALEYLSULFATHIAZOLE [1] | | | −92.507 | −8.0 |
| CHEMBL2104058 | CARPRAZIDIL | | | −69.079 | −7.9 |
| CHEMBL2104945 | SULFACLORAZOLE | | | −92.009 | −7.9 |
| CHEMBL2105145 | NIFURALIDE | | | −83.434 | −7.9 |
| CHEMBL4211107 | ATB-346 | Phase2 | | −60.796 | −7.8 |
| CHEMBL2104449 | SARAKALIM | | | −63.011 | −7.8 |
| CHEMBL26630 | SULFATROXAZOLE | | Endothelin receptor ET-A | −76.825 | −7.8 |
| CHEMBL12552 | BIMAKALIM [1] | | | −79.030 | −7.7 |
| CHEMBL3989584 | IDRALFIDINE | | | −64.196 | −7.7 |
| CHEMBL1481457 | GLYSOBUZOLE | | Aldehyde dehydrogenase 1A1 | −91.008 | −7.6 |
| CHEMBL2106717 | BUTADIAZAMIDE | | | −84.490 | −7.6 |
| CHEMBL2106363 | ISOSULPRIDE | | | −60.047 | −7.5 |
| CHEMBL2106536 | CYCLAZODONE | | | −67.971 | −7.5 |
| CHEMBL2105110 | LAMTIDINE | | | −71.290 | −7.4 |
| CHEMBL2105508 | SULFATROZOLE | | | −67.271 | −7.4 |
| CHEMBL593262 | PARA-NITROSULFATHIAZOLE [1] | | | −83.527 | −7.4 |
| CHEMBL608806 | COCAETHYLENE | Phase1 | Monoclonal antibody (mAb) 2E2 | −60.968 | −7.2 |
| CHEMBL2106208 | ETASULINE | | | −91.878 | −7.1 |
| CHEMBL2104333 | ICODULINUM | | | −83.698 | −7.0 |
| CHEMBL2104022 | ACLANTATE | | | −65.024 | −6.9 |
| CHEMBL2105108 | ISAGLIDOLE | | | −85.089 | −6.9 |

| | | | | | |
|---|---|---|---|---|---|
| CHEMBL2104120 | CLANTIFEN | | | −85.470 | −6.5 |
| CHEMBL2106134 | DALBRAMINOL [1] | | | −69.252 | −6.5 |
| CHEMBL2105025 | IQUINDAMINE | | | −83.311 | −6.2 |
| CHEMBL2105256 | PRANOSAL | | | −61.508 | −6.2 |
| CHEMBL2106311 | LEUCINOCAINE | | | −64.900 | −6.2 |
| CHEMBL2106760 | FLUTONIDINE | | | −88.413 | −6.2 |
| CHEMBL55812 | OLPADRONIC ACID | | Trypanosoma brucei rhodesiense | −69.980 | −5.9 |
| CHEMBL62382 | METHYLSELENOCYSTEINE | Phase1 | Kynurenine-oxoglutarate transaminase I | −80.956 | nd [5] |

[1]Potential drugs for SARS-CoV-2 $M^{pro}$ in Ref [1]. [2]Repurposing drugs for SARS-CoV-2. [3]Nucleoside triphosphate analogs. [4]No data (compound contains an unsupported boron atom). [5]No data (compound contains an unsupported selenium atom).

**Table S2.** Hit compounds obtained by combining the AutoDock Vina and rDock virtual screening results using the ChEMBL database.

| ChEMBL ID | Target | AutoDock Vina Score (kcal/mol) |
|---|---|---|
| CHEMBL4246021 | Citrobacter freundii | −13.9 |
| CHEMBL3905018 | Serine/threonine-protein kinase mTOR | −13.5 |
| CHEMBL1256418 | Receptor protein-tyrosine kinase erbB-2 | −13.4 |
| CHEMBL600492 | | −13.3 |
| CHEMBL4590896 | Plasmodium falciparum | −13.1 |
| CHEMBL4552365 | MDA-MB-231 | −13.0 |
| CHEMBL4457485 | Streptococcus gordonii | −13.0 |
| CHEMBL3944416 | Tyrosine-protein kinase SYK | −13.0 |
| CHEMBL3940079 | MAP kinase p38 alpha | −12.9 |
| CHEMBL4557444 | Porphyromonas gingivalis | −12.9 |
| CHEMBL3698432 | Tyrosine-protein kinase SYK | −12.9 |
| CHEMBL3641104 | Serine/threonine-protein kinase B-raf | −12.8 |
| CHEMBL4434852 | TIGK | −12.8 |
| CHEMBL3238193 | Protein-tyrosine phosphatase 1B | −12.7 |

| | | |
|---|---|---|
| CHEMBL4444544 | Stimulator of interferon genes protein | −12.7 |
| CHEMBL3958000 | Tyrosine-protein kinase BTK | −12.7 |
| CHEMBL2263277 | Xanthomonas campestris | −12.7 |
| CHEMBL3939320 | Apoptosis regulator Bcl-X | −12.6 |
| CHEMBL3694020 | Serine/threonine-protein kinase B-raf | −12.6 |
| CHEMBL1993470 | SN12C | −12.6 |
| CHEMBL178947 | Telomerase reverse transcriptase | −12.6 |
| CHEMBL4090562 | Tyrosine-protein kinase receptor UFO | −12.6 |
| CHEMBL3898710 | Tyrosine-protein kinase SYK | −12.6 |
| CHEMBL1213559 | | −12.6 |
| CHEMBL3774987 | | −12.6 |
| CHEMBL3794423 | | −12.6 |
| CHEMBL3357287 | Bcr/Abl fusion protein | −12.5 |
| CHEMBL4579682 | MDA-MB-231 | −12.5 |
| CHEMBL572749 | Micrococcus flavus | −12.5 |
| CHEMBL2037222 | Nerve growth factor receptor Trk-A | −12.5 |
| CHEMBL4561646 | Plasmodium falciparum | −12.5 |
| CHEMBL580070 | Plasmodium falciparum | −12.5 |
| CHEMBL3401277 | Rattus norvegicus | −12.5 |
| CHEMBL450266 | Serine/threonine-protein kinase AKT | −12.5 |
| CHEMBL3974105 | Tyrosine-protein kinase SYK | −12.5 |
| CHEMBL243919 | Tyrosine-protein kinase TIE-2 | −12.5 |
| CHEMBL1199180 | | −12.5 |
| CHEMBL3775025 | | −12.5 |
| CHEMBL4116218 | | −12.5 |
| CHEMBL11725 | Ancylostoma ceylanicum | −12.4 |
| CHEMBL275918 | Ancylostoma ceylanicum | −12.4 |
| CHEMBL409500 | Autotaxin | −12.4 |
| CHEMBL3309842 | Bacillus subtilis | −12.4 |
| CHEMBL272121 | BaF3 | −12.4 |
| CHEMBL2048347 | Beta-secretase 1 | −12.4 |
| CHEMBL3899126 | Casein kinase II alpha/beta | −12.4 |

| | | |
|---|---|---|
| CHEMBL4112597 | Coagulation factor IX | −12.4 |
| CHEMBL3922109 | MAP kinase p38 alpha | −12.4 |
| CHEMBL298873 | Purinergic receptor P2Y12 | −12.4 |
| CHEMBL120478 | Rattus norvegicus | −12.4 |
| CHEMBL3684453 | Serine/threonine-protein kinase AKT | −12.4 |
| CHEMBL561268 | Staphylococcus aureus | −12.4 |
| CHEMBL1095051 | Staphylococcus epidermidis | −12.4 |
| CHEMBL3964386 | Tyrosine-protein kinase SYK | −12.4 |
| CHEMBL297941 | | −12.4 |
| CHEMBL448196 | | −12.4 |
| CHEMBL548692 | | −12.4 |
| CHEMBL3823355 | KB | −12.3 |
| CHEMBL4171392 | Klebsiella pneumoniae | −12.3 |
| CHEMBL3897917 | MAP kinase p38 alpha | −12.3 |
| CHEMBL1160003 | Nucleic Acid | −12.3 |
| CHEMBL1092387 | Peroxisome proliferator-activated receptor gamma | −12.3 |
| CHEMBL4580177 | Plasmodium falciparum | −12.3 |
| CHEMBL3219913 | Plasmodium falciparum 3D7 | −12.3 |
| CHEMBL4441513 | Porphyromonas gingivalis | −12.3 |
| CHEMBL50724 | Purinergic receptor P2Y12 | −12.3 |
| CHEMBL454210 | Serine/threonine-protein kinase AKT2 | −12.3 |
| CHEMBL3669598 | Serine/threonine-protein kinase PAK 4 | −12.3 |
| CHEMBL3933037 | Serine/threonine-protein kinase RAF | −12.3 |
| CHEMBL1972753 | SN12C | −12.3 |
| CHEMBL278606 | Staphylococcus aureus | −12.3 |
| CHEMBL3641673 | Tyrosine-protein kinase SYK | −12.3 |
| CHEMBL1205686 | | −12.3 |
| CHEMBL3306679 | | −12.3 |
| CHEMBL4595227 | | −12.3 |
| CHEMBL587109 | | −12.3 |
| CHEMBL1170512 | Acetyl-CoA carboxylase | −12.2 |
| CHEMBL3651843 | ALK tyrosine kinase receptor | −12.2 |

| | | |
|---|---|---|
| CHEMBL1779459 | Aspergillus oryzae | −12.2 |
| CHEMBL3668756 | Complement factor D | −12.2 |
| CHEMBL2316899 | Dual specificity protein phosphatase 3 | −12.2 |
| CHEMBL3604941 | Epidermal growth factor receptor erbB1 | −12.2 |
| CHEMBL218416 | Escherichia coli | −12.2 |
| CHEMBL218721 | Escherichia coli | −12.2 |
| CHEMBL4167104 | Hepatocyte growth factor receptor | −12.2 |
| CHEMBL3116482 | HERG | −12.2 |
| CHEMBL3978486 | Insulin-like growth factor I receptor | −12.2 |
| CHEMBL3921959 | MAP kinase p38 alpha | −12.2 |
| CHEMBL3964270 | MAP kinase p38 alpha | −12.2 |
| CHEMBL4514182 | Porphyromonas gingivalis | −12.2 |
| CHEMBL1256422 | Receptor protein-tyrosine kinase erbB-2 | −12.2 |
| CHEMBL3680780 | Rho-associated protein kinase 2 | −12.2 |
| CHEMBL3641102 | Serine/threonine-protein kinase B-raf | −12.2 |
| CHEMBL1970631 | SN12C | −12.2 |
| CHEMBL4520286 | Tyrosine-protein kinase ITK/TSK | −12.2 |
| CHEMBL4071242 | Tyrosine-protein kinase receptor UFO | −12.2 |
| CHEMBL4161666 | | −12.2 |
| CHEMBL3905470 | Apoptosis regulator Bcl-X | −12.1 |
| CHEMBL3949706 | Apoptosis regulator Bcl-X | −12.1 |
| CHEMBL258469 | Autotaxin | −12.1 |
| CHEMBL3309844 | Bacillus subtilis | −12.1 |
| CHEMBL3663195 | DNA gyrase | −12.1 |
| CHEMBL2206745 | Dual specificity phosphatase Cdc25B | −12.1 |
| CHEMBL488574 | Furin | −12.1 |
| CHEMBL579430 | HepG2 | −12.1 |
| CHEMBL3661012 | Insulin-like growth factor I receptor | −12.1 |
| CHEMBL4583938 | L1210 | −12.1 |
| CHEMBL1213154 | MAP kinase p38 alpha | −12.1 |
| CHEMBL3935885 | MAP kinase p38 alpha | −12.1 |
| CHEMBL3946614 | MAP kinase p38 alpha | −12.1 |

| | | |
|---|---|---|
| CHEMBL574054 | Micrococcus flavus | −12.1 |
| CHEMBL2386806 | MOLM-13 | −12.1 |
| CHEMBL4216323 | Mycobacterium tuberculosis | −12.1 |
| CHEMBL4534583 | Plasmodium falciparum | −12.1 |
| CHEMBL4555672 | Plasmodium falciparum | −12.1 |
| CHEMBL4582854 | Plasmodium falciparum | −12.1 |
| CHEMBL534053 | Plasmodium falciparum | −12.1 |
| CHEMBL1277758 | Plasmodium yoelii nigeriensis | −12.1 |
| CHEMBL1256423 | Receptor protein-tyrosine kinase erbB-2 | −12.1 |
| CHEMBL1969834 | Serine/threonine-protein kinase Aurora-A | −12.1 |
| CHEMBL3689913 | Serine/threonine-protein kinase B-raf | −12.1 |
| CHEMBL3909544 | Serine/threonine-protein kinase Sgk1 | −12.1 |
| CHEMBL1807708 | SK-MEL-28 | −12.1 |
| CHEMBL1997404 | SN12C | −12.1 |
| CHEMBL22723 | Staphylococcus aureus | −12.1 |
| CHEMBL235941 | Staphylococcus aureus | −12.1 |
| CHEMBL364874 | Staphylococcus aureus | −12.1 |
| CHEMBL4090946 | Tyrosine-protein kinase BTK | −12.1 |
| CHEMBL4110832 | Tyrosine-protein kinase BTK | −12.1 |
| CHEMBL4113946 | Tyrosine-protein kinase BTK | −12.1 |
| CHEMBL3698429 | Tyrosine-protein kinase SYK | −12.1 |
| CHEMBL3969554 | Tyrosine-protein kinase SYK | −12.1 |
| CHEMBL1195620 | | −12.1 |
| CHEMBL1199215 | | −12.1 |
| CHEMBL1739538 | | −12.1 |
| CHEMBL2178272 | | −12.1 |
| CHEMBL2237058 | | −12.1 |
| CHEMBL3297827 | | −12.1 |
| CHEMBL3963814 | Apoptosis regulator Bcl-X | −12.0 |
| CHEMBL2314162 | Carbonic anhydrase II | −12.0 |
| CHEMBL168162 | Corticotropin releasing factor receptor 1 | −12.0 |
| CHEMBL3658931 | DNA gyrase | −12.0 |

| | | |
|---|---|---|
| CHEMBL2206742 | Dual specificity phosphatase Cdc25B | −12.0 |
| CHEMBL3604938 | Epidermal growth factor receptor erbB1 | −12.0 |
| CHEMBL3980253 | GroEL/GroES | −12.0 |
| CHEMBL4205192 | MAP kinase ERK1 | −12.0 |
| CHEMBL3972567 | MAP kinase p38 alpha | −12.0 |
| CHEMBL4061895 | MGC-803 | −12.0 |
| CHEMBL573890 | Micrococcus flavus | −12.0 |
| CHEMBL4579172 | Mycobacterium tuberculosis | −12.0 |
| CHEMBL3627920 | Nucleic Acid | −12.0 |
| CHEMBL32741 | P388 | −12.0 |
| CHEMBL4447611 | Plasmodium falciparum | −12.0 |
| CHEMBL4522857 | Plasmodium falciparum | −12.0 |
| CHEMBL4566305 | Plasmodium falciparum | −12.0 |
| CHEMBL2430796 | Pseudomonas aeruginosa | −12.0 |
| CHEMBL120321 | Rattus norvegicus | −12.0 |
| CHEMBL2315364 | Rattus norvegicus | −12.0 |
| CHEMBL1256417 | Receptor protein-tyrosine kinase erbB-2 | −12.0 |
| CHEMBL1256429 | Receptor protein-tyrosine kinase erbB-2 | −12.0 |
| CHEMBL4579941 | Sepiapterin reductase | −12.0 |
| CHEMBL3692214 | Serine/threonine-protein kinase Aurora-B | −12.0 |
| CHEMBL3694024 | Serine/threonine-protein kinase B-raf | −12.0 |
| CHEMBL1971789 | SN12C | −12.0 |
| CHEMBL2018735 | TCF4/beta-catenin | −12.0 |
| CHEMBL4090260 | Toll-like receptor 4 | −12.0 |
| CHEMBL499445 | Trypanosoma brucei brucei | −12.0 |
| CHEMBL4210185 | Tyrosine kinase non-receptor protein 2 | −12.0 |
| CHEMBL1076474 | Tyrosine-protein kinase LCK | −12.0 |
| CHEMBL4100738 | Tyrosine-protein kinase receptor UFO | −12.0 |
| CHEMBL3891553 | Tyrosine-protein kinase SYK | −12.0 |
| CHEMBL3923486 | Tyrosine-protein kinase SYK | −12.0 |
| CHEMBL3678955 | Vascular endothelial growth factor receptor 2 | −12.0 |
| CHEMBL3972455 | Vascular endothelial growth factor receptor 2 | −12.0 |

| | |
|---|---|
| CHEMBL1086295 | −12.0 |
| CHEMBL1231573 | −12.0 |
| CHEMBL2207914 | −12.0 |
| CHEMBL3306628 | −12.0 |
| CHEMBL3765281 | −12.0 |
| CHEMBL3990330 | −12.0 |
| CHEMBL4104974 | −12.0 |
| CHEMBL4116805 | −12.0 |
| CHEMBL4203222 | −12.0 |
| CHEMBL4571014 | −12.0 |

## Reference

[1] Tsuji M. Potential anti-SARS-CoV-2 drug candidates identified through virtual screening of the ChEMBL database for compounds that target the main coronavrius protease. FEBS Open Bio **2020**, 10, 995−1004.
